# Clinical trial highlights: Dopamine cell-replacement therapies

**DOI:** 10.1177/1877718X251397277

**Published:** 2025-11-25

**Authors:** Saeed Kayhanian, Roger A Barker

**Affiliations:** 1 Department of Clinical Neurosciences, University of Cambridge, Cambridge, UK; 2 Department of Neurosurgery, Cambridge University Hospitals, Cambridge, UK; 3 Cambridge Stem Cell Institute, Cambridge, UK; 4 Department of Neurology, Cambridge University Hospitals, Cambridge, UK

**Keywords:** Cell therapy, Parkinson's disease, stem cell, clinical trials, neurosurgery

## Abstract

Parkinson's disease is a common neurodegenerative disorder, which is characterised by motor features, many of which relate to the loss of the dopaminergic nigrostriatal pathway. The use of grafted cells to replace the lost dopaminergic neurons as a therapy for Parkinson's has been explored since the 1980s, with mixed clinical outcomes. Much of the heterogeneity in outcomes has been related to the major problems with the cell source for these trials, being derived from human fetal brain tissue. It is, however, now possible to derive authentic midbrain dopamine cells from human pluripotent stem cells and several first-in-human clinical trials are now underway to explore this approach.

## Plain language summary

Dopamine cell replacement therapy is a potential treatment for Parkinson's disease, which aims to treat the disease by directly replacing some of the cells which have been lost in the brain as part of the pathology. This article presents a summary and discussion of the current clinical trial landscape of dopamine cell replacement therapy for Parkinson's disease.

## Introduction

Parkinson's disease (PD) is a common neurodegenerative disorder, which is characterised by motor features, many of which relate to the loss of the dopaminergic nigrostriatal pathway. This pathway takes its origin from the A9 nigral dopaminergic neurons of which there are between 400–500,000 in the human midbrain and the first motor features of PD typically emerge when around half of these cells are lost.^1^

Drugs targeting this dopaminergic pathway have been used with success since the 1960s but, although effective in early disease, over time L-dopa medications cause their own motor complications including on-off fluctuations and dyskinesias, while the dopamine agonists have other side effects, most notably neuropsychiatric problems. There is therefore a need for better dopamine therapies targeting this pathway and while Deep Brain Stimulation (DBS) and more recently MRI focused ultrasound lesioning new approaches, along with subcutaneous pump infusions of L-dopa or apomorphine, are being used for advanced PD, another way to do this would be by replacing the lost cells through dopamine cell replacement.^2^

The feasibility of this approach in people with Parkinson's disease (PwP) was first explored in pioneering studies undertaken in Lund in the 1980s and 1990s, initially attempted using autologous cells from the adrenal medulla but soon switching to using allogeneic dopaminergic cells taken from developing human fetal ventral mesencephalon (hfVM), derived from donated tissue from terminated pregnancies. The hfVM cells were implanted into the host striatum in a stereotactic-guided neurosurgical procedure. The results of the first case series using hfVM tissue were encouraging, demonstrating the safety of this approach and evidence of graft survival, as assessed using ^18^Fluoro-dopa (F-DOPA) PET imaging.^3^ The clinical outcomes, however, were mixed, with isolated cases demonstrating significant improvement (including being able to come off dopaminergic medication completely for many years) but others showing no benefit. This approach, using hfVM grafted into the putamen, went on to be tested as part of two NIH-funded double-blind placebo controlled trials in the 1990s, which then reported in 2001 and 2003. In both these trials, the primary outcome was not met and a significant number of patients developed side-effects, including graft-induced dyskinesias (GIDs).^4,5^

The discouraging results of these two trials led to a re-evaluation within the field and a pause in further trials using this approach. In 2006, an international workshop was set up to explore all the available evidence in the field and to attempt to identify why some patients appeared to have benefited significantly from fetal dopamine cell replacement whilst others had not.^6^ Through a limited meta-analysis of published studies, several factors were identified as being associated with a better outcome after hfVM transplantation, including younger recipients with less advanced PD, ensuring adequate hfVM tissue was grafted to ensure that at least 100,000 dopaminergic nigral neurons survived, and adequate immunosuppression post-grafting.^7,8^ In addition, it was also clear that for this field to move on, some better understanding of GIDs was needed. Several theories were put forward regarding the development of this complication, including uneven dopamine innervation across the putamen post grafting; excessive dopamine production by the transplants; or the co-transplantation of the adjacently developing serotonin-producing neurons which lack a dopamine transporter system to inactivate any dopamine released by these cells.

As a result, a new trial using hfVM tissue was devised and then funded through the EU (TransEuro - NCT01898390). However, this trial encountered major problems with hfVM supply, meaning that the trial could not proceed as planned, and only 21 surgeries were conducted over a 3 year period, with 87 planned surgeries having to be cancelled because of issues of tissue supply.^9,10^ As such a more reliable source of dopamine cells is needed if this therapy is ever to become a clinical reality and this possibility arose with the development of protocols for making authentic midbrain dopamine cells from human pluripotent stem cells in 2011/12.

The first such trial of stem-cell derived dopaminergic cell replacement therapy for PwP started in 2015 and 15 trials to date have commenced – see below. We searched both ClinicalTrials.gov and WHO clinical trials registries for any trials using dopamine cells derived from stem cells as a treatment for Parkinson's disease. Most trials are still ongoing and the field is at an early stage of development and so knowing whether they are safe and can work is unproven.^11^

## Human embryonic stem cell-derived dopaminergic cell trials (Table 1)

Human embryonic stem cell (hESC)-derived cells use the pluripotent cells derived from a single human embryo's inner cell mass at day 4/5 post-fertilisation as the original cell source. These cells can be expanded in-vitro and several such hES cell lines have now been derived at a clinical-grade. These pluripotent cells can then be differentiated to dopaminergic precursor cells in-vitro. This approach allows for an ‘off-the-shelf’ therapy, with cell products that can be cryopreserved and produced in large quantities to a clinical-standard, with the advantage that samples from each batch can be tested for quality control. This approach leads to an allogeneic product with maturation of the dopamine cells post implantation and the need for some form of immunosuppressive regimen post-grafting.

**Table 1.** Summary of embryonic stem cell-derived trials.

Trial number	Sponsor	Start date	Number of participants	Status
NCT03119636	Chinese Academy of Sciences	2015	50	Unknown
NCT04802733	BlueRock Therapeutics	2021	12	Completed
NCT05635409	Region Skane	2022	8	Recruitment completed
NCT05887466	S. Biomedics	2023	12	Ongoing
IRCT20160704028786N2	Royan Institute	2025	4	Ongoing

Title: Safety and Efficacy Study of Human ESC-derived Neural Precursor Cells in the Treatment of Parkinson's Disease

Phase: I/II

Start date: 2015

Status: Unknown

Clinical Trial ID: NCT03119636

Sponsor: Chinese Academy of Sciences

Country: China

Study design: Open label trial administering a single dose of dopaminergic neural precursor cells by stereotactic intra-striatal injection. This study aimed to enrol 50 patients, aged between 50–80y with later stage PD (Hoehn and Yahr [HY] Stage 3 or 4). The primary outcome was safety, measuring the number and nature of adverse events.

Comments: This is the first clinical trial that attempted to use stem-cell derived dopaminergic grafts in PwP. There has been no clinical data released.

Title: Phase 1 Study To Assess the Safety and Tolerability of Human Embryonic Stem Cell-Derived Midbrain Dopamine Neuron Cell Therapy (MSK-DA01) For Advanced Parkinson's Disease

Phase: I

Start date: 2021

Status: Completed

Clinical Trial ID: NCT04802733

Sponsor: BlueRock Therapeutics

Country: USA/Canada

Study design: Open label trial administering an ESC-derived dopaminergic progenitor product (Bemdaneprocel) by intra-putamenal injection. Two cohorts were recruited to test doses of 1.8 M and 5.4 M cells, with a total of 12 patients in the trial. The trial recruited PwP aged between 50–78y with advanced PD. The primary outcome was safety and tolerability at 12 months post-transplantation, with secondary outcomes measuring cell survival by 18F-DOPA PET imaging and changes in motor score, as measured by the Unified Parkinson's Disease Rating Scale (UPDRS) at 1 and 2 years post-transplant.

Comments: This trial completed recruitment in 2022 and has reported that the product is well tolerated in both high and low dose cohorts at 24-months post-transplantation. They report also a mean reduction of 23 points on the UPDRS Part III score in the ‘OFF’ state, compared with a reduction of 8.6 in the low dose cohort, suggesting a dose-dependent motor response.^12^ On the basis of this trial, BlueRock Therapeutics/Bayer have recently announced their intention to advance the Bemdaneprocel product to a Phase III registrational trial, with a placebo-controlled design.^13^

Title: A Trial to Determine the Safety and Tolerability of Transplanted Stem Cell Derived Dopamine Neurons to the Brains of Individuals With Parkinson's Disease (STEM-PD)

Phase: I

Start date: 2022

Status: Ongoing, recruitment completed

Clinical Trial ID: NCT05635409

Sponsor: Region Skane

Country: Sweden/UK

Study design: Open label trial administering an ESC-dopaminergic progenitor product (STEM-PD) through intra-putamental injection. Two cohorts of 4 patients were recruited to test two doses of 7 M and 14 M cells (n = 8 total). The trial recruited PwP aged between 50–75y, with moderate PD (HY stage 2–3 in OFF state). The primary outcome is safety, as assessed by the number and nature of adverse events at 12 months post-transplantation, with secondary outcomes exploring changes in clinical features, including motor aspects of the condition as recorded through the UPDRS Part III.

Comments: This trial completed recruitment and grafting in 2024 and the data collection is ongoing.

Title: Study to Evaluate the Safety and Efficacy of ESC-derived Dopamine Progenitor Cell Therapy in PD Patients

Phase: I/II

Start date: 2023

Status: Ongoing

Clinical Trial ID: NCT05887466

Sponsor: S. Biomedics

Country: South Korea

Study design: Open label trial administering an ESC-dopaminergic progenitor product (A9-DPC) by intra-putamenal injection. Two cohorts of 6 patients have been recruited to test two doses of 3.15 M and 6.3 M cells. The trial is recruiting PwP aged between 50–75y with advanced PD (HY3–4 in the OFF state). The primary outcome is safety at 24 months, measuring the occurrence and nature of treatment-emergent adverse events after administration of the cell product. The trial reported a favourable safety profile at 12 months.^14^

Title: The Assessment of Safety and Feasibility of Intra-Striatal Transplantation of Human Embryonic stem cell-derived Dopaminergic Progenitors (DopaCell) in Parkinson's disease: A Multicenter Phase I

Start date: 2025

Status: Ongoing

Clinical Trial ID: IRCT20160704028786N2

Sponsor: Royan Institute

Country: Iran

Study design: Open label trial administering an ESC-dopaminergic progenitor product (DopaCell) by intra-striatal injection. The trial intends to recruit 4 patients to test a single dose of 10 M cells. The trial is recruiting patients aged between 30–70y at HY Stage 3 in the OFF state. The primary outcome is safety and feasibility, as measured by the number and nature of adverse events at 12 months post-transplantation.

## Human induced pluripotent stem-cell derived dopaminergic cell trials (Table 2)

Human induced pluripotent stem cells (iPSCs) are pluripotent cells that are derived directly from somatic cells. Somatic cells are induced into a pluripotent state by the introduction of a cocktail of transcription factors in-vitro. These pluripotent cells can then be further differentiated into dopaminergic neurons in-vitro. Since iPSCs can be derived directly from patients’ own cells (e.g., from fibroblasts taken in a skin biopsy or circulating blood cells), this allows the potential for autologous cell transplants, which theoretically removes the need for immunosuppression regimens. This highly personalised autologous therapeutic approach however is not without concerns, given the manufacturing costs and the risk that the cells may do less well given they are derived from the patient with PD. Several clinical-grade iPSC lines have now been generated and banked, so trials of allogeneic iPSC-derived dopaminergic cells are underway, as well as autologous approaches.

**Table 2.** Summary of induced pluripotent stem cell (iPSC)-derived trials.

Trial number	Sponsor	Start date	Number of participants	Cell source	Status
NCT06687837	McLean Hospital, Massachusetts General Hospital	2017	1	Autologous iPSCs from fibroblast sample	Completed
UMIN000033564	Kyoto University Hospital	2018	7	Allogeneic iPSCs	Completed
NCT06344026	Aspen Neuroscience	2024	9	Autologous iPSCs from skin cells	Recruitment completed
NCT06422208	McLean Hospital	2024	6	Autologous iPSCs from blood sample	Ongoing
NCT06608355	iRegene Therapeutics	2024	6	Allogeneic iPSCs	Ongoing
NCT06167681	iRegene Therapeutics	2024	40	Allogeneic iPSCs	Ongoing
NCT06482268	University of California, San Diego	2024	7	Allogeneic iPSCs	Ongoing
NCT06753331	Sumitomo Pharma America	2025	23	Allogeneic iPSCs	Ongoing
NCT06778265	Shanghai UniXell Biotechnology	2025	12	Autologous iPSCs (source not specified)	Ongoing
NCT06821529	iCamuno Biotherapeutics	2025	12	Autologous iPSCs (source not specified)	Ongoing
NCT07106021	Kenai Therapeutics	2025	12	Allogeneic iPSC	Ongoing

Title: Treating Parkinson's Disease Through Transplantation of Autologous Stem Cell-Derived Dopaminergic Neurons

Phase: I/II

Start date: 2017 (n = 1), 2024

Status: Ongoing

Clinical Trial ID: NCT06687837

Sponsor: McLean Hospital, Massachusetts General Hospital

Country: USA

Study design: Open label trial administering dopaminergic progenitor cells derived from autologous iPSCs, produced from patient fibroblast samples. Cells will be transplanted by intra-putamenal injection. 8 patients will be recruited and two doses (8 M and 16 M cells) will be tested. The trial will recruit PwP aged between 45–80y with advanced PD, HY3–4 in the OFF state. The primary outcome is safety as measured through recording the incidence and severity of adverse events 2 years following transplantation.

Comments: This clinical trial that started in 2024 builds on the results of a single case using the same approach, which was published in 2020.^15^ In this single case, there were no serious adverse events at 24 months and a possible suggestion of clinical benefit, at least subjectively, using the PDQ-39 patient questionnaire.

Title: Kyoto Trial to Evaluate the Safety and Efficacy of iPSC-derived dopaminergic progenitors in the treatment of Parkinson's Disease

Phase: I/II

Start date: 2018

Status: Completed

Clinical Trial ID: UMIN000033564

Sponsor: Kyoto University Hospital

Country: Japan

Study design: Open label trial administering allogeneic-iPSC derived dopaminergic precursor cells by intra-putamenal injection. 7 patients were recruited at two doses of 4.2–5.2 M (n = 3) and 10.6–11 M cells (n = 4). The trial recruited PwP aged between 50–70y with advanced PD (HY3–4 in the OFF state). The primary outcome was safety and the presence or absence of graft expansion at 24 months following transplantation.

Comments: This trial has completed, reporting no serious adverse events and an average reduction of 9.5 points in the UPDRS Part III score.^16^ Of note, this trial used allogeneic-iPSCs derived from a donor with a HLA-genotype which matches 17% of the Japanese population, however participants were not specifically HLA-matched as part of the trial and immunosuppression was used post-grafting.^17^ The cell product in this trial (also known as CT1-DAP001 or DSP-1083) has been taken forward by Sumitomo Pharma Company in two further studies with open label and sham-controlled designs (see below).

Title: Phase 1/​2a Study of ANPD001 in Parkinson Disease (ASPIRO)

Phase: I/II

Start date: 2024

Status: Ongoing

Clinical Trial ID: NCT06344026

Sponsor: Aspen Neuroscience

Country: USA

Study design: Open label trial administering dopaminergic progenitor cells derived from autologous iPSCs, produced from patients’ own skin cells. 9 patients have been be recruited for two cohorts receiving 10 M or 15–20 M cells by intra-putamenal injection. The trial recruited PwP aged between 45–80y with advanced PD (HY3–4 in the OFF state). The primary outcome is safety at 2 years following transplantation, measured by the incidence and severity of adverse events.

Comments: This trial has completed recruitment and grafting in January 2025, reporting no serious adverse events to this point.^18^

Title: Autologous iPSC-Derived Dopamine Neuron Transplantation for Parkinson's Disease

Phase: I

Start date: 2024

Status: Ongoing

Clinical Trial ID: NCT06422208

Sponsor: McLean Hospital

Country: USA

Study design: Open label trial administering dopaminergic progenitor cells derived from autologous iPSCs, generated from the patients’ own blood cells. 6 patients will be recruited and all receive the same number of cells (dose not disclosed) by intra-putamenal injection. The trial will recruit PwP aged between 55–80y with a diagnosis of PD for at least 5 years. The primary outcome is safety at 2 years following transplantation, as measured by the incidence and severity of adverse events.

Title: The Safety, Tolerability and Preliminary Efficacy of NouvNeu001 for Early-onset Parkinson's Disease

Phase: I

Start date: 2024

Status: Ongoing

Clinical Trial ID: NCT06608355

Sponsor: iRegene Therapeutics

Country: China

Study design: Open label trial administering dopaminergic progenitor cells derived from allogeneic iPSCs. 6 patients will be recruited for single dose (not disclosed) by intra-striatal injection. The trial will recruit PwP aged between 18–70y with an age of disease onset <50y and HY stage 2–4 in the OFF state. The primary outcome is safety at 96 weeks following transplantation, as measured by the incidence and severity of adverse events.

Title: The Safety, Tolerability and Efficacy of NouvNeu001 for Parkinson's Disease

Phase: I/II

Start date: 2024

Status: Ongoing

Clinical Trial ID: NCT06167681

Sponsor: iRegene Therapeutics

Country: China

Study design: Open label trial administering dopaminergic progenitor cells derived from allogeneic iPSCs. 40 patients will be recruited for single dose (not disclosed) by intra-striatal injection. The trial will recruit PwP aged between 50–75y with a HY stage 2–4 in the OFF state. The primary outcome is safety at 24 and 48 weeks post-transplantation and motor function, as measured by change in UPDRS Part III score, at 24 weeks post-transplantation.

Comments: The NovNeu001 allogeneic iPSC-derived dopaminergic product is being tested in parallel to the Phase 1 study (above). There is also a difference in the cohorts being recruited, with the Phase 1 study recruiting PwP who had early onset disease, compared with a more typical age of onset in the Phase 1/2 study.

Title: Transplantation of Human iPS Cell-derived Dopaminergic Progenitors (CT1-DAP001) for Parkinson's Disease (Phase I/​II)

Phase: I/II

Start date: 2024

Status: Ongoing

Clinical Trial ID: NCT06482268

Sponsor: University of California, San Diego

Country: USA

Study design: Open label trial administering dopaminergic progenitor cells derived from allogeneic iPSCs. 7 patients will be recruited for a single dose of 8.4–10.8 M cells by intra-putamenal injection. The trial will recruit PwP aged between 40–75y with a HY stage ≥2 in the OFF state. The primary outcome is safety, as measured by the incidence and severity of treatment emergent adverse events and presence or absence of graft expansion on F-DOPA imaging at 24 months after transplantation.

Title: A Multicenter Study to Evaluate Safety, Tolerability, and Clinical Responses of DSP-1083 Into Subjects with Parkinson's Disease

Phase: I

Start date: 2025

Status: Ongoing

Clinical Trial ID: NCT06753331

Sponsor: Sumitomo Pharma America

Country: USA

Study design: A two cohort study administering dopaminergic progenitor cells derived from allogeneic iPSCs by intraputamenal injection. A sentinel cohort (n = 3) will receive 5.4 M cells and second cohort (n = 20) will be randomised in a 1:1 ratio to receive 5.4 M cells or sham-surgery (bilateral, partial thickness burr hole procedure). The trial will recruit PwP aged between 40–70y with a HY stage ≥3 in the OFF state. Participants in the sentinel cohort must be of Asian ethnicity but participants in the second cohort will be recruited from any ethnicity. The primary outcomes are safety, measured number the number and nature of adverse events, and clinical response, measured by neuropsychiatric assessments and change in F-DOPA uptake on PET imaging at 104 weeks post-transplantation.

Title: An Exploratory Clinical Study of UX-DA001 in Subjects with Idiopathic Parkinson's Disease

Phase: I

Start date: 2025

Status: Ongoing

Clinical Trial ID: NCT06778265

Sponsor: Shanghai UniXell Biotechnology

Country: China

Study design: Open label trial administering dopaminergic progenitor cells derived from autologous iPSCs. 12 patients will be recruited for two dose cohorts (dose not disclosed) by intra-putamenal injection. The trial will recruit PwP aged between 50–75y with a HY stage 3–4 in the OFF state. The primary outcome is safety, as measured by incidence and severity of adverse events at 4 weeks and 2 years following transplantation.

Title: Stereotactic Intracerebral Injection of IPSC-DAPs in Patients with Parkinson's Disease

Phase: 1

Start date: 2025

Status: Ongoing

Clinical Trial ID: NCT06821529

Sponsor: iCamuno Biotherapeutics

Country: China

Study design: Open label trial administering dopaminergic progenitor cells derived from autologous iPSCs. 12 patients will be recruited for a dose of 8 M cells administered by intra-putamenal injection. The trial will recruit PwP aged between 39–75y with a HY stage 2–4 in the OFF state. The primary outcome is safety, measured by incidence and severity of adverse events at 12 months following transplantation.

Title: A Study to Assess Safety and Efficacy of Surgical Implant of RNDP-001 in Patients With Idiopathic Parkinson's Disease

Phase: 1b/2a

Start date: 2025

Status: Ongoing

Clinical Trial ID: NCT07106021

Sponsor: Kenai Therapeutics

Country: USA

Study design: Open label trial administering dopaminergic progenitor cells derived from allogeneic iPSCs. 12 patients will be recruited for three doses (unspecified) administered by intra-putamenal injection. The trial will recruit PwP aged between 45–75y. The primary outcome is safety, measured by incidence and severity of adverse events at 15 months following transplantation.

## Summary

The field of dopamine cell replacement therapy as a treatment for Parkinson's has now entered a new phase as the first trials using stem cell derived neuronal precursor cells are now being published, with several more trials ongoing. The results from these trials will help in the design of bigger phase 2/3 trials looking for efficacy which in turn will help demonstrate whether these therapies offer a competitive benefit over what is already available in the treatment of people with Parkinson's around dopaminergic therapies. These latter therapies encompass a range of pump treatments as well as neurosurgical interventions and, while they work well in many patients, they do bring their own problems and issues especially around complications and the need for ongoing follow-up. Dopamine cell therapies offer an advantage in this respect in that they are a one-off treatment, albeit with a period of immunosuppression in many cases. However, unlike dopaminergic pump therapies and DBS, the cells cannot be modulated to optimise efficacy and thus getting the correct dose range will be critical. In addition, the costs of any such cell therapy will need to compete with those being offered for these more advanced PD treatments – but this may prove less than imagined given the short duration and scale up of the current differentiation protocols.^19^ However, it is possible that if such transplants are shown to work in moderately advanced patients (the current target group – see below) then going earlier in the disease course may be possible and with this come cost savings in terms of anti-PD therapies and the complications they bring, as well as social costs by allowing individuals to function at a higher level, including remaining in work.

As is clear from our outline of the ongoing trials, these therapies are currently being trialled in people with moderately advanced disease who are looking at some more invasive intervention in the next few years. This group has been chosen for ethical reasons, given these treatments are being trialled for the first time in Parkinson's and thus one cannot legitimately ask early-stage patients to have this intervention and it would be too hazardous for those with more advanced disease. This recruitment of moderately advanced people with Parkinson's, does allow for the efficacy of a dopamine therapy to be more easily detected given they have fluctuating disease often with complex dopaminergic drug regimens. However, choosing the right patients is still not easy as these dopamine cell therapies can take years to work. Thus, finding the ‘sweet spot for grafting’ is not straightforward as one cannot offer it to patients who will need some sort of more advanced therapy in the next 1–2 years, as they do not have the time to wait for the grafted cells to differentiate, innervate and then work.

In the next round of dopamine cell therapy trials, the need for sham/imitation surgery will need to be considered which varies around the world in terms of whether this is mandated or not by the regulators. There is a significant potential for a placebo effect with a surgical intervention of this sort but we, and others, have argued that the use of a placebo arm is not needed, given; (i) the size of response one needs to see for the therapy to be therapeutically competitive; (ii) the ability to objectively visualise the graft using PET imaging and the degree of normalisation of striatal dopamine post grafting and (iii) the problems of maintaining a blind in the modern era with electronic health records and patient access.^20^ As such the need for sham surgery is likely to be driven as much by the FDA and other regulators as by the trialists themselves.

Finally deciding who is optimal for these therapies is still unknown and while there are some obvious criteria, such as response to oral dopaminergic treatments, there are many unknowns and that extends to the degree of immunosuppression needed in allogeneic grafting (both the regimen and duration of it). Such questions can only be answered as the number of trials increases and ideally with some sort of central register which would allow for some form of meta-analysis, as was attempted for human fetal DA transplants over 10 years ago.^8^ This may help to highlight subgroups of patients who particularly benefit from such intervention and enable more targeted trial designs in the future. Such data will also show the size and sustainability of any clinical effect and while this therapy is not disease modifying in terms of slowing down the disease process, it does have the potential to dramatically change the natural history of treated Parkinson's through reducing the need for patients to take oral anti-PD drugs and the complications that they bring with them. As such dopamine cell therapies offer great hope to people with Parkinson's but at present it remains a hope as they have yet to be proven to work well and competitively for this condition.
